# Ureidopyrazine Derivatives: Synthesis and Biological Evaluation as Anti-Infectives and Abiotic Elicitors

**DOI:** 10.3390/molecules22101797

**Published:** 2017-10-23

**Authors:** Ghada Bouz, Martin Juhás, Pavlína Niklová, Ondřej Janďourek, Pavla Paterová, Jiří Janoušek, Lenka Tůmová, Zuzana Kovalíková, Petr Kastner, Martin Doležal, Jan Zitko

**Affiliations:** Faculty of Pharmacy in Hradec Kralove, Charles University, Heyrovskeho 1203, Hradec Kralove 50005, Czech Republic; juhasm@faf.cuni.cz (M.J.); niklovp@faf.cuni.cz (P.N.); jando6aa@faf.cuni.cz (O.J.); pavla.paterova@fnhk.cz (P.P.); janousj2@faf.cuni.cz (J.J.); tumova@faf.cuni.cz (L.T.); kovalikz@faf.cuni.cz (Z.K.); kastner@faf.cuni.cz (P.K.); dolezalm@faf.cuni.cz (M.D.)

**Keywords:** abiotic elicitors, anti-infectives, callus culture, ester, *Mycobacterium tuberculosis*, pyrazinoic acid, ureidopyrazine

## Abstract

Tuberculosis (TB) caused by *Mycobacterium tuberculosis* (*Mtb*) has become a frequently deadly infection due to increasing antimicrobial resistance. This serious issue has driven efforts worldwide to discover new drugs effective against *Mtb*. One research area is the synthesis and evaluation of pyrazinamide derivatives as potential anti-TB drugs. In this paper we report the synthesis and biological evaluations of a series of ureidopyrazines. Compounds were synthesized by reacting alkyl/aryl isocyanates with aminopyrazine or with propyl 5-aminopyrazine-2-carboxylate. Reactions were performed in pressurized vials using a CEM Discover microwave reactor with a focused field. Purity and chemical structures of products were assessed, and the final compounds were tested in vitro for their antimycobacterial, antibacterial, and antifungal activities. Propyl 5-(3-phenylureido)pyrazine-2-carboxylate (compound **4**, MIC*_Mtb_* = 1.56 μg/mL, 5.19 μM) and propyl 5-(3-(4-methoxyphenyl)ureido)pyrazine-2-carboxylate (compound **6**, MIC*_Mtb_* = 6.25 μg/mL, 18.91 μM) had high antimycobacterial activity against *Mtb* H37Rv with no in vitro cytotoxicity on HepG2 cell line. Therefore **4** and **6** are suitable for further structural modifications that might improve their biological activity and physicochemical properties. Based on the structural similarity to 1-(2-chloropyridin-4-yl)-3-phenylurea, a known plant growth regulator, two selected compounds were evaluated for similar activity as abiotic elicitors.

## 1. Introduction

**Figure 1:**
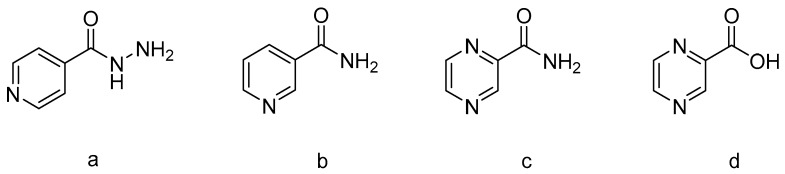
The chemical structure of (**a**) INH; (**b**) nicotinamide; (**c**) PZA; and POA (**d**).

Tuberculosis (TB) is a common infection that had been successfully treated with appropriate first line anti-TB drugs, including isoniazid (INH), rifampicin, pyrazinamide (PZA), and ethambutol [1]. Yet in the last few years, this curable infection has become a frequently deadly illness due to the raising issue of antimicrobial resistance (AMR) [1]. AMR includes multi-drug resistant TB (MDR-TB), when the bacteria is resistant to both INH and rifampicin, and extensively drug resistant TB (XDR-TB), when the bacteria is resistant to INH, rifampicin, fluoroquinolones, and one of the three parenteral second line drugs (amikacin, kanamycin, or capreomycin) [2]. Despite the fact that the annual number of deaths due to TB has fallen from the year 2000 till today, TB remains one of the top ten causes of death worldwide and the leading cause of death from infectious diseases [3]. According to the World Health Organization (WHO) annual report, there were 10.4 million new cases of TB worldwide in 2015, out of which 480,000 cases were MDR-TB [3]. The number of deaths attributed to TB in that year was 1.4 million, whether due to inaccessibility to treatment or treatment failure [2]. It is estimated that by 2050 deaths due to AMR will reach 10 million deaths/year in comparison to 8.2 million deaths/year due to all types of cancer combined if no measures are implemented to stop resistance spread [4]. In 2015 alone, the number of deaths of TB due to AMR was 250,000 [3]. The serious issue of AMR has driven efforts worldwide to find new therapeutic drugs *Mycobacterium tuberculosis* (*Mtb*) bacteria are sensitive to. An ongoing research area is the synthesis and evaluation of PZA derivatives as potential anti-TB drugs [5,6,7,8,9,10]. PZA is considered to be an analogue of nicotinamide and its chemical structure is closely related to INH (Figure 1). PZA plays an important role in conventional (drug-sensitive) TB treatment regimen as it shortens the duration of therapy from 9–12 months to 6 months [11]. It is converted to its active form, pyrazinoic acid (POA, Figure 1), intracellularly in acidic pH by a hydrolytic enzyme known as pyrazinamidase (PZase) encoded by the *Mtb* bacterium itself [12]. Several new specific mechanisms were identified by which PZA or POA exerts its anti-TB activity. Those include interference with ribosomal protein S1 (RpsA) [12], inhibition of quinolinic acid phosphoribosyl transferase (QAPRTase) [13], inhibition of aspartate decarboxylase [14], and inhibition of Fatty acid synthase I (FAS I) [15,16]. The knowledge of such new targets will help to design new potentially active PZA derivatives that may overcome the issue of AMR. 

In this paper, we focus on the design, synthesis, and anti-infective evaluation of ureidopyrazine derivatives. In general, urea derivatives have shown wide range of pharmacological activities, including hypoglycemic, anti-cancer, anticonvulsant, antiviral, and antimicrobial activities, as reviewed elsewhere [17]. For instance, *N*-alkylurea hydroxamic acids showed potent antibacterial activity against both Gram-positive and Gram-negative bacteria by inhibiting peptide deformylase (PDF) that is essential for bacterial growth [18]. Furthermore, one compound from this series (compound **19**) has been already proved to be a Chk-1 kinase inhibitor with anticancer activity [19], yet have not been evaluated for any anti-infective property. Title compounds of this study were evaluated for their antimycobacterial, antibacterial, and antifungal activities in vitro. Two of the prepared compounds, **8** and **18**, were further assessed as plant abiotic elicitors since similar pyrazinecarboxylic acid derivatives were proved to exert such activity. The chemical structures of the latter two compounds also resemble that of a commercially available plant growth promotor, 1-(2-chloropyridin-4-yl)-3-phenylurea (for its structure readers may refer to Section 2.2.5).

## 2. Results and Discussion

### 2.1. Chemistry

#### 2.1.1. Compounds **1**–**9**

**Scheme 1:**
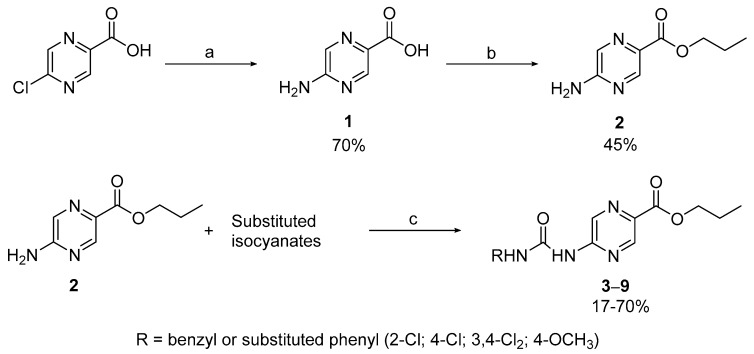
Synthetic procedures of compounds **1**–**9**. *Reagents and Conditions:* (**a**) NH_3_ (25% aq.sol), MW: 100 °C, 30 min, 80 W; (**b**) esterification with propanol, H_2_SO_4_, MW: 100 °C, 1 h, 80 W; (**c**) hexane, MW: 120 °C, 1 h, 80 W.

The starting 5-aminopyrazine-2-carboxylic acid (**1**) was prepared by reacting 5-chloropyrazine-2-carboxylic acid with an aqueous solution of ammonia to replace the chlorine atom with amino group (Scheme 1, step a). Then, it was esterified (Fischer esterification) with propanol in the presence of catalytic amounts of concentrated sulfuric acid to yield propyl 5-aminopyrazine-2-carboxylate (**2**) (Scheme 1, step b). The obtained ester was then reacted with six different aromatic substituted isocyanates in hexane (Scheme 1, step c), resulting in six different aryl substituted ureidopyrazine propyl esters (Group B). The esterification step preceded the introduction of the urea moiety to the molecule in order to prevent potential unwanted side-reactions between the isocyanates and the free carboxylic acid moiety that results in amide formation [20], and also to prevent possible decarboxylation at the high reaction temperature. The most biologically active ester **4** was then hydrolyzed to **5** in methanol and potassium carbonate as a base to compare the biological activity of the ester to the corresponding free carboxylic acid.

#### 2.1.2. Compounds **10**–**20**

**Scheme 2:**
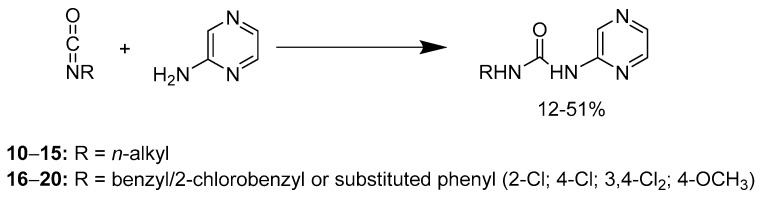
Synthetic procedure of compounds **10**–**20**. *Reagents and Conditions*: hexane, MW: 120 °C, 1 h, 80 W.

Aminopyrazine was reacted with five different alkyl substituted isocyanates (Group C) and six different substituted phenyl or benzyl isocyanates (Group D) in one step reaction, in the presence of hexane as solvent, to yield the corresponding ureidopyrazine derivatives lacking the ester moiety of group B compounds (Scheme 2). We also attempted reacting 3-chloropyrazin-2-amine and 6-chloro-pyrazin-2-amine with different isocyanates under same conditions but no product was detected.

All chemical reactions were performed in pressurized test vials in a CEM Discover microwave reactor with a focused field. The over-pressurized system of microwave test tubes is essential to achieve higher temperatures than the boiling points of solvents (propanol and hexane). Moreover, the closed system prevents the escape of ammonia from the reaction mixture.

Final ureido derivatives were purified using flash chromatography. They were isolated as solids compounds of white to yellow color, in yields ranging from 12% to 70% of chromatographically pure products. Then, they were characterized by their melting points, ^1^H- and ^13^C-NMR spectra, IR spectroscopy, and elemental analysis. The acquired analytical data fully supported the corresponding proposed structures.

### 2.2. Biological Activity

#### 2.2.1. Antimycobacterial Activity Evaluation against *Mycobacterium tuberculosis*, *Mycobacterium kansasii*, and Mycobacterium *avium*

All prepared compounds, including the starting acid and ester that lack the urea moiety, were screened for in vitro activity against *Mtb* H37R_v_, *Mycobacterium kansasii* (*M. kansasii*) and *Mycobacterium avium* (*M. avium*) using a Microplate Alamar Blue Assay [21]. Antimycobacterial activity results were expressed as minimum inhibitory concentration (MIC) in μg·mL^−1^ in comparison with INH as standard (Table 1).

Five of the twenty prepared compounds had anti-TB activity within the range of tested concentrations. Based on the results of the biological evaluation (Table 1), we can conclude that in this series the ester moiety is important for anti-TB activity. When compound **4** (MIC*_Mtb_* = 1.56 μg/mL, 5.19 μM) (log *P* = 1.62) was hydrolyzed to compound **5** (MIC*_Mtb_* > 100 μg/mL, >387.23 μM) (log *P* = 0.54), it lost its biological activity, suggesting that the free carboxylic moiety significantly reduced the lipophilicity and hence could have impaired penetration through the highly lipophilic mycobacterial cell wall. Lipophilicity is an important aspect of activity against *Mtb*. The five active compounds had log *P* values ranging from 1.11 to 2.18. Compounds with either lower or higher lipophilicity had diminished activity. However, lipophilicity is not the only determinant of anti-TB activity; compounds **7** and **8** share the same log *P* value, yet compound **8** is active while **7** is not. We also found that non-substituted phenyl (compound **4**) or 4-monosubstituted phenyl (compound **6**) derivatives of ureidopyrazine esters had higher anti-TB activity than other substituents. Interestingly, 4-chloro substitution on the phenyl core resulted in active compounds in the series with ester moiety (Compound **8**, MIC*_Mtb_* = 25 μg/mL), as well as in the series lacking the ester moiety (compound **18**, MIC*_Mtb_* = 12.5 μg/mL). When comparing the chemical structure and anti-TB activity of compounds **3** and **4**, we found that the introduction of a -CH_2_- bridge resulted in loss of biological activity. Aryl substituted ureidopyrazine esters (Group B) had better activity than the corresponding non-ester compounds of the same substituent (Group D). When comparing the non-ester compounds (**10**–**20**), alkyl substituted ureidopyrazines (Group C) had inferior activity to the aryl derivatives (Group D). Among the aliphatic series (Group C), compound **13** (MIC*_Mtb_* = 25 μg/mL, 99.86 μM) exerted the highest anti-TB activity. This result is consistent with pervious findings in our research group with *N*-Alkyl-3-(alkylamino)-pyrazine-2-carboxamides of 6–8 carbon alkyl chain having the best activity [23]. Since the starting acid and ester had no urea moiety, we can infer that urea is an important pharmacophore for the activity of such designed compounds. Compounds **4** (MIC*_Mtb_* = 1.56 μg/mL, 5.19 μM) and **6** (MIC*_Mtb_* = 6.25 μg/mL, 18.91 μM) are suitable for further structural modifications that might improve their anti-TB activity and physicochemical properties. Compound **6** (MIC*_M.kansasii_* = 25 μg/mL, 75.67 μM) and compound **13** (MIC*_M.kansasii_* = 25 μg/mL, 89.79 μM) were the only two compounds with moderate activity against *M. kansasii*. None of the tested compounds showed activity against *M. avium.*

#### 2.2.2. Antimycobacterial Activity Evaluation against *Mycobacterium smegmatis* and *Mycobacterium aurum*

In order to compare the activity of prepared compounds on fast growing mycobacteria to that on previously mentioned slow growing *Mtb*, *M. kansasii*, and *M. avium*, this complementary screening was conducted. This test has several advantages since *M. smegmatis* and *M. aurum* are avirulent surrogate organisms [24]. Unlike slow growing mycobacteria that cause serious courses of infection in humans, *M. smegmatis* and *M. aurum* result in soft tissue infections in humans [25,26,27]. The two latter mycobacteria also have similar antibiotic susceptibility profile to *Mtb* [28]. Microplate Alamar Blue assays with INH, rifampicin, and ciprofloxacin as standards was used in performing this test. None of the tested compounds exerted activity against the mentioned fast growing mycobacteria up to the highest tested concentration of 125 µg/mL for compounds **9**, **17**, **20**, 250 µg/mL for compounds **8**, **14**, **19**, and 500 µg/mL for the remaining compounds (Supplementary Materials, Table S1), suggesting that those compounds active against *Mtb* might work through a pathway not shared among all species of mycobacteria. 

#### 2.2.3. Antibacterial and Antifungal Activity Evaluation

Most compounds were tested in vitro for their biological activity against eight common bacterial strains and eight fungal strains of clinical importance using standard methods [29]. The test excluded compounds **3**, **6**, **7**, **9**, and **18** due to precipitation in the testing media upon dilution. None of the tested compounds exerted antibacterial or antifungal activity up to the highest tested concentrations of 125 µM for compounds **4**, **8**, **16**, 250 µM for compound **14**, and 500 µM for the remaining compounds µM (Supplementary Materials, Tables S2 and S3).

#### 2.2.4. In Vitro Cytotoxicty Assays

**Figure 2:**
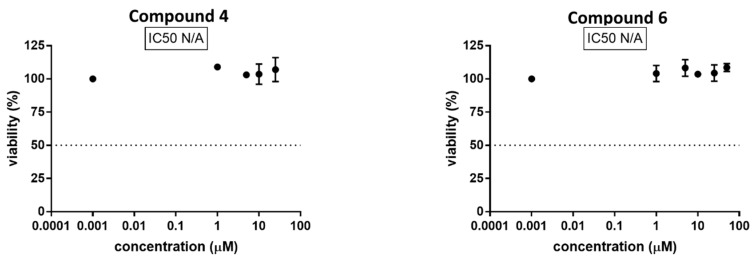
Cytotoxic effect of different concentrations of the tested substances on HepG2 cells.

TB treatment regimens are known to carry a risk of hepatotoxicity [30]. Therefore, it is important when developing new anti TB drugs test them for this crucial side effect. The most active compounds, **4** and **6**, were further evaluated for any possible cytotoxicity. For this screening, standard hepatic cell line HepG2 (hepatocellular carcinoma) was used, and results were expressed by the inhibitory concentration required to decrease the viability of cell population to 50% (IC_50_) compared to a control of 100% cell viability. The used a CellTiter 96 assay is based on the reduction of tetrazolium dye MTS in living cells to formazan, which is then determined colorimetrically. Due to low solubility at higher concentrations in cell culture medium, it was difficult to determine the exact IC_50_ of compounds **4** and **6**. However, it can be stated that those two compounds were nontoxic at their highest tested concentrations (Table 2, Figure 2).

Data points from precipitated concentrations are not shown.

#### 2.2.5. Plant Growth Regulation Activity Evaluation

**Figure 3:**
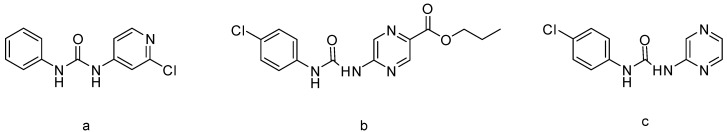
The chemical structure of (**a**) 1-(2-chloropyridin-4-yl)-3-phenylurea; (**b**) compound **8**; and (**c**) compound **18**.

Previous studies showed that derivatives of pyrazine-2-carboxylic acid used as elicitors were able to increase secondary metabolites production in in vitro plant cultures. Compounds **8** and **18** structurally resemble a plant growth regulator, 1-(2-chloropyridin-4-yl)-3-phenylurea (Figure 3). This chemical promotes cell division and growth, leading to an increase in fruit size. Its use has been approved in the USA on kiwi and grapes [32]. Based on those two facts, the effect of compounds **8** and **18** on rutin production in *Fagopyrum esculentum* var. Bamby callus culture was assessed.

Full numerical data regarding the content of rutin in callus cultures of *F. esculentum* after treatment with compounds **8** and **18** separately can be found in Supplementary Materials, Table S4. For compound **8**, the highest rutin levels, 0.80 (70.2% increase) and 0.83 (76.6% increase) µg·g^−1^ DW, were reached 6 and 12 h respectively post treatment in comparison with control (24 K). Gradual decrease in rutin content was observed after 48 and 72 h of compound **8** application. On the other hand, compound **18** resulted in a more significant increase in rutin level 6 h post application (115% increase) when compared to control (24 K, control after 24 h). After 12 and 24 h of elicitor application, rutin content is increased only about 12.8% and 21.2%. The two compounds decreased rutin production in similar fashion after 48 and 72 h; however, compound **18** further decreased rutin content in comparison with control (168 K, control after 168 h).

These results indicate that the two proposed elicitors are able to increase rutin production in callus culture of *Fagopyrum esculetum* var. Bamby. This increase in rutin production level is mainly affected by the time of elicitor application. According to literature, secondary metabolite production in general is affected by various factors, including the type of elicitor, its concentrations, and the time of administration. In our previous studies we declared the positive effect of various concentrations of pyrazinecarboxamide derivatives on the flavonoids and flavonolignans production in callus and suspension cultures of *Ononis arvensis* and *Silybum marianum* [33,34] and the current results support ureidopyrazines as well.

## 3. Materials and Methods

### 3.1. General Information

All chemicals were of reagent or higher grade of purity. They were purchased from Sigma-Aldrich (Steinheim, Germany), unless stated otherwise. Progress of reactions was checked by using Merck Silica 60 F_254_ TLC plates (Merck, Darmstadt, Germany) with UV detection using 254 nm wavelength. Microwave assisted reactions were performed in a CEM Discover microwave reactor with a focused field (CEM Corporation, Matthews, NC, USA) connected to an Explorer 24 autosampler (CEM Corporation) and this equipment was running under CEM’s SynergyTM software for setting and monitoring the conditions of reactions. The temperature of the reaction mixture was monitored by internal infrared sensor. All obtained products were purified by preparative flash chromatograph CombiFlash^®^ Rf (Teledyne Isco Inc., Lincoln, NE, USA). The type of elution was gradient, using the mixture of hexane (LachNer, Neratovice, Czech Republic) and ethyl acetate (Penta, Prague, Czech Republic) as mobile phase. Silica gel (0.040–0.063 nm, Merck, Darmstadt, Germany) was used as the stationary phase. NMR spectra were recorded on Varian VNMR S500 (Varian, Palo Alto, CA, USA) at 500 MHz for ^1^H and 125 MHz for ^13^C. Chemical shifts were reported in ppm (δ) and were referred indirectly to tetramethylsilane via signal of solvent (2.49 for ^1^H and 39.7 for ^13^C in DMSO-*d*_6_; 7.26 for ^1^H and 77.2 for ^13^C in CDCl_3_). Infrared spectra were recorded with spectrometer FT-IR Nicolet 6700 (Thermo Scientific, Waltham, MA, USA) using attenuated total reflectance (ATR) methodology on germanium crystal. Elemental analysis was performed on vario MICRO cube Element Analyzer (Elementar Analysensysteme, Hanau, Germany). All values regarding elemental analyses are given as percentages. Melting points were determined in open capillary on Stuart SMP30 melting point apparatus (Bibby Scientific Limited, Staffordshire, UK) and are uncorrected. Yields are expressed as percentages of theoretical yields and refer to the isolated products (chromatographically pure) after all purification steps. Theoretical lipophilicity parameters log *P* was calculated by algorithms of program CS ChemBioDraw Ultra 16.0 (CambridgeSoft, Cambridge, MA, USA).

### 3.2. Synthesis

#### 3.2.1. Compounds **1** and **2**

The starting 5-chloropyrazine-2-carboxylic acid (317 mg, 2 mmol) was converted to 5-aminopyrazine-2-carboxylic acid (**1**) by substitution reaction with 25% (*m*/*m*) aqueous solution of ammonia (3 mL). The reaction was carried out 10 mL microwave pressurized vials with stirring (reaction temperature: 100 °C, reaction time: 30 min, power output: 80 W). The reaction was repeated 20 times to yield reasonable quantity of the starting acid. Once the reaction was completed, the vials content was put onto Petri dish and heated above a water bath with intermittent stirring until a dry solid was obtained (ammonium salt of the product). To get the free acid form, the ammonium salt was dissolved in water and drop-wise acidified with 10% hydrochloric acid to reach pH of 4. The mixture was then left to cool down in room temperature for 5 min then kept in the fridge for 15 min. The formed free acid crystals were filtered off by filtration paper with suction and left to dry overnight. After it was dried, the resulting 5-aminopyrazine-2-carboxylic acid (**1**) was esterified in several microwave pressurized vials; 3 mL of anhydrous propanol and 2 drops of concentrated sulfuric acid were added to 278 mg (2 mmol) of compound **1** in each vial. The esterification was carried out in microwave reactor (reaction temperature: 100 °C, reaction time: 1 h, power output: 80 W). The completion of reaction was monitored by TLC in system hexane/ethyl acetate (EtOAc) (1:3). The ester was then purified by flash chromatography using gradient elution 40 to 100% EtOAc in hexane.

#### 3.2.2. Compounds **3**–**9**

The resulted propyl 5-aminopyrazine-2-carboxylate (**2**) (360 mg, 2 mmol) was reacted with different aryl substituted isocyanates (2.2 mmol) in hexane as solvent (3 mL). The reaction was carried out in 10 mL microwave pressurized vials with stirring (reaction temperature: 120 °C, reaction time: 1 h, power output: 80 W). Reactions were monitored by TLC in system hexane/EtOAc (1:3). The reaction mixture was adsorbed to silica and then purified by flash chromatography using gradient elution 0 to 100% EtOAc in hexane. 

Compound **4** (130 mg, 0.5 mmol) was hydrolyzed to **5** by base catalyzed hydrolysis, using anhydrous potassium carbonate (3 g, 20 mmol) as base and methanol (approximately 20 mL) as solvent. The reaction mixture was stirred and heated to 85 °C under reflux in an oil bath for approximately 6 h. Potassium carbonate was then filtered off and the mixture was acidified with 10% hydrochloric acid to reach a pH of 3. The solution was evaporated and the product washed with water to remove any remnants of potassium carbonate and then left to dry. 

#### 3.2.3. Compounds **10**–**20**

In a 10 mL microwave pressurized vial, commercially available aminopyrazine (190 mg, 2 mmol) was reacted with different aryl/alky substituted isocyanates (2.2 mmol) in hexane as solvent (3 mL) with a magnetic stirrer. The reaction proceeded in a microwave reactor with stirring (reaction temperature: 120 °C, reaction time: 1 h, power output: 80 W). Reaction process was monitored by TLC in system hexane/EtOAc (1:3). The reaction mixture was adsorbed to silica and then purified by flash chromatography using gradient elution 20 to 100% EtOAc in hexane for alkyl substituted ureidopyrazines and 0 to 70% EtOAc in hexane for aryl substituted ureidopyrazines. N.B.: Alkyl substituted ureidopyrazines **10**–**14** needed some time to solidify after the evaporation of solvents after the flash chromatography.

### 3.3. Analytical Data of the Prepared Compounds

*5-Aminopyrazine-2-carboxylic acid* (**1**). Light pinkish solid. Yield 70%; m.p. 238–245 °C; IR (ATR-Ge, cm^−1^): 3316 (-NH-), 1643 (-C=O), 1621, 1591 (arom.); ^1^H-NMR (DMSO-*d*_6_) δ 12.58 (s, 1H,-COOH), 8.51 (d, *J* = 1.3 Hz, 1H, arom.), 7.91 (d, *J* = 1.3 Hz, 1H, arom.), 7.27 (s, 2H, -NH_2_). ^13^C-NMR (DMSO-*d*_6_) δ 165.8, 157.4, 145.7, 131.9, 130.8. Elemental analysis found: C, 42.82%; H, 3.51%; N, 29.64%. Calculated for C_5_H_5_N_3_O_2_ (MW 139.11): C, 43.17%; H, 3.62%; N, 30.21%.

*Propyl 5-aminopyrazine-2-carboxylate* (**2**). Yellow solid. Yield 45%; m.p. 135–138 °C; IR (ATR-Ge, cm^−1^): 3317 (-NH-), 1722 (-C=O), 1661, 1584 (arom.); ^1^H-NMR (DMSO-*d*_6_) δ 8.53 (d, *J* = 1.3 Hz, 1H, arom.), 7.91 (d, *J* = 1.3 Hz, 1H, arom.), 7.32 (s, 2H, -NH_2_), 4.16 (t, *J* = 6.7 Hz, 2H, -CH_2_-), 1.73–1.62 (m, 2H, -CH_2_-), 0.93 (t, *J* = 7.4 Hz, 3H, -CH_3_). ^13^C-NMR (DMSO-*d*_6_) δ 164.4, 157.5, 145.7, 132.3, 130.2, 65.8, 21.8, 10.5. Elemental analysis found: C, 53.44%; H, 6.26%; N, 22.71%. Calculated for C_8_H_11_N_3_O_2_ (MW 181.20): C, 53.03%; H, 6.12%; N, 23.19%.

*Propyl 5-(3-benzylureido)pyrazine-2-carboxylate* (**3**). White solid. Yield 65%; m.p. 190.5–193.3 °C; IR (ATR-Ge, cm^−1^): 3329 (-NH-), 1717 (-C=O, ester), 1705 (-C=O, urea), 1625, 1539, 1471, 1456 (arom.); ^1^H-NMR (CDCl_3_) δ 10.67 (s, 1H, urea), 9.36 (s, 1H, urea), 8.82 (d, *J* = 1.3 Hz, 1H, pyrazine), 8.54–8.50 (m, 1H, pyrazine), 7.40–7.28 (m, 4H, arom.), 7.32–7.26 (m, 1H, arom.), 4.62 (d, *J* = 5.8 Hz, 2H, -OCH_2_-), 4.43–4.33 (m, 2H, -NCH_2_-), 1.92–1.78 (m, 2H, -CH_2_-), 1.04 (t, *J* = 14.8, 7.4 Hz, 3H, -CH_3_). ^13^C-NMR (CDCl_3_) δ 163.9, 155.8, 151.2, 142.7, 138.9, 138.3, 136.0, 135.7, 128.8, 128.7, 127.5, 127.5, 127.4, 67.6, 67.4, 44.7, 44.0, 22.0, 10.4. Elemental analysis found: C, 60.73%; H, 5.66%; N, 17.58%. Calculated for C_16_H_18_N_4_O_3_ (MW 314.35): C, 61.14%; H, 5.77%; N, 17.82%.

*Propyl 5-(3-phenylureido)pyrazine-2-carboxylate* (**4**). White solid. Yield 17%; m.p. 217.9–219.9 °C; IR (ATR-Ge, cm^−1^): 3302 (-NH-), 1721 (-C=O, ester), 1704 (-C=O, urea), 1604, 1557, 1540, 1506, 1499 (arom.); ^1^H-NMR (DMSO-*d*_6_) δ 10.02 (s, 1H, urea), 9.69 (s, 1H, urea), 9.13–9.08 (m, 1H, pyrazine), 8.90–8.81 (m, 1H, pyrazine), 7.54–7.49 (m, 1H, arom.), 7.36–7.29 (m, 2H, arom.), 7.06–7.02 (m, 2H, arom.), 4.28–4.19 (m, 2H, -OCH_2_-), 1.72–1.68 (m, 2H, -CH_2_-), 0.96 (t, 3H, -CH_3_).^13^C-NMR (DMSO-*d*_6_) δ 163.6, 151.4, 144.0, 138.6, 136.0, 134.7, 129.1, 123.3, 119.1, 66.6, 21.8, 10.5. Elemental analysis found: C, 60.43%; H, 5.59%; N, 18.38%. Calculated for C_15_H_16_N_4_O_3_ (MW 300.32): C, 59.99%; H, 5.37%; N, 18.66%.

*Propyl 5-(3-Phenylureido)pyrazine-2-carboxylic acid* (**5**). White solid. Yield 35%; m.p. 228–229.3 °C; IR (ATR-Ge, cm^−1^): 3569 (-NH-), 1728 (-C=O, acid), 1685 (-C=O, urea), 1596, 1567, 1552, 1500 (arom.); ^1^H-NMR (DMSO-*d*_6_) δ 13.27 (s, 1H, -COOH), 9.98 (s, 1H, urea), 9.73 (s, 1H, urea), 9.12 (d, *J* = 1.4 Hz, 1H, pyrazine), 8.87 (d, *J* = 1.4 Hz, 1H, pyrazine), 7.52 (d, *J* = 8.0 Hz, 2H, arom.), 7.33 (t, *J* = 7.8 Hz, 2H, arom.). ^13^C-NMR (DMSO-*d*_6_) δ 165.1, 151.5, 151.2, 144.0, 138.6, 136.8, 134.4, 129.1, 123.2, 119.1. Elemental analysis found: C, 62.88%; H, 5.50%; N, 24.16%. Calculated for C_12_H_10_N_4_O_3_ (MW 258.24): C, 63.15%; H, 5.30%; N, 24.55%.

*Propyl 5-(3-(4-methoxyphenyl)ureido)pyrazine-2-carboxylate* (**6**). White solid. Yield 39%; m.p. 189–193 °C; IR (ATR-Ge, cm^−1^): 3333 (-NH-), 1718 (-C=O, ester), 1702 (-C=O, urea), 1610, 1546, 1508, 1404 (arom.), 1231(-C-O); ^1^H-NMR (CDCl_3_) δ 11.05 (s, 1H, urea), 10.87 (s, 1H, urea), 8.90 (d, *J* = 1.3 Hz, 1H, pyrazine), 8.56 (s, 1H, pyrazine), 7.54–7.42 (m, 2H, arom.), 6.97–6.84 (m, 2H, arom.), 4.39 (t, *J* = 6.9 Hz, 2H, -OCH_2_-), 3.84 (s, 3H, -OCH_3_), 1.92–1.80 (m, 2H, -CH_2_-), 1.06 (t, *J* = 7.4 Hz, 3H, -CH_3_). ^13^C-NMR (CDCl_3_) δ 163.8, 156.5, 153.4, 150.8, 142.4, 135.9, 135.8, 130.2, 122.0, 114.3, 67.4, 55.5, 22.0, 10.3. Elemental analysis found: C, 58.58%; H, 5.62%; N, 16.62%. Calculated for C_16_H_18_N_4_O_4_ (MW 330.34): C, 58.17%; H, 5.49%; N, 16.96%.

*Propyl 5-(3-(2-chlorophenyl)ureido)pyrazine-2-carboxylate* (**7**). White solid. Yield 28%; m.p. 195–197 °C; IR (ATR-Ge, cm^−1^): 3567 (-NH-), 1720 (-C=O, ester), 1689 (-C=O, urea), 1636, 1591, 1549, 1511, 1473 (arom.); ^1^H-NMR (CDCl_3_) δ 11.86 (s, 1H, urea), 10.90 (s, 1H, urea), 8.86 (d, *J* = 1.4 Hz, 1H, pyrazine), 8.52 (d, *J* = 1.4 Hz, 1H, pyrazine), 8.32 (dd, *J* = 1.5, 8.3 Hz, 1H, arom.), 7.38 (dd, *J* = 1.5, 8.0 Hz, 1H, arom.), 7.31–7.23 (m, 1H, arom.), 7.09–6.99 (m, 1H, arom.), 4.39 (t, *J* = 6.9 Hz, 2H, -OCH_2_-), 1.94–1.81 (m, 2H. -CH_2_-), 1.06 (t, *J* = 7.4 Hz, 3H, -CH_3_). ^13^C-NMR (CDCl_3_) δ 163.7, 153.3, 150.4, 142.4, 136.3, 135.4, 134.9, 129.3, 127.8, 124.6, 123.4, 121.3, 67.5, 22.1, 10.4. Elemental analysis found: C, 54.12%; H, 4.59%; N, 16.58%. Calculated for C_15_H_15_ClN_4_O_3_ (MW 334.76): C, 53.82%; H, 4.52%; N, 16.74%.

*Propyl 5-(3-(4-chlorophenyl)ureido)pyrazine-2-carboxylate* (**8**). White solid. Yield 57%; m.p. 240.6–243.8 °C; IR (ATR-Ge, cm^−1^): 3328 (-NH-), 1720 (-C=O, ester), 1704 (-C=O, urea), 1606, 1540, 1493, 1471, 1454 (arom.); ^1^H-NMR (DMSO-*d*_6_) δ 10.17 (s, 1H, urea), 10.02 (s, 1H, urea), 9.11–8.99 (m, 1H, pyrazine), 8.92–8.84 (m, 1H, pyrazine), 7.93 (d, *J* = 2.5 Hz, 1H, arom.), 7.56 (d, *J* = 8.8 Hz, 1H, arom.), 7.45 (dd, *J* = 8.8, 2.5 Hz, 2H, arom.), 4.26 (t, *J* = 6.6 Hz, 2H, -OCH_2_-), 1.78–1.67 (m, 2H, -CH_2_-), 0.96 (t, *J* = 7.4 Hz, 3H, -CH_3_). ^13^C-NMR (DMSO-*d*_6_) δ 163.6, 151.4, 151.3, 143.9, 137.6, 136.2, 134.7, 128.9, 126.9, 120.7, 66.6, 21.7, 10.5. Elemental analysis found: C, 53.39%; H, 4.39%; N, 16.53%. Calculated for C_15_H_15_ClN_4_O_3_ (MW 334.76): C, 53.82%; H, 4.52%; N, 16.74%.

*Propyl 5-(3-(2,3-dichlorophenyl)ureido)pyrazine-2-carboxylate* (**9**). White solid. Yield 70%; m.p. 242.6–243.9 °C; IR (ATR-Ge, cm^−1^): 3336 (-NH-), 1705 (-C=O, ester), 1655 (-C=O, urea), 1612, 1537, 1481, 1378 (arom.); ^1^H-NMR (DMSO-*d*_6_) δ 10.17 (s, 1H, urea), 10.02 (s, 1H, urea), 9.11–8.99 (m, 1H, pyrazine), 8.92–8.84 (m, 1H, pyrazine), 7.93 (d, *J* = 2.5 Hz, 1H, arom.), 7.56 (d, *J* = 8.8 Hz, 1H, arom.), 7.45 (dd, *J* = 8.8, 2.5 Hz, 1H, arom.), 4.26 (t, *J* = 6.6 Hz, 2H, -OCH_2_-), 1.78–1.67 (m, 2H, -CH_2_-), 0.96 (t, *J* = 7.4 Hz, 3H, -CH_3_). ^13^C-NMR (DMSO-*d*_6_) δ 163.6, 151.4, 151.0, 143.9, 138.8, 136.3, 134.8, 131.4, 130.9, 124.7, 120.3, 119.3, 66.7, 21.7, 10.5. Elemental analysis found: C, 48.65%; H, 3.56%; N, 14.84%. Calculated for C_15_H_14_Cl_2_N_4_O_3_ (MW 369.20): C, 48.80%; H, 3.82%; N, 15.18%.

*1-Propyl-3-(pyrazin-2-yl)urea* (**10**). Light yellow solid. Yield 36%; m.p. 104.6–107.1 °C; IR (ATR-Ge, cm^−^^1^): 3274 (-NH-), 2963 (-CH_2_-), 1694 (-C=O), 1586, 1536, 1499 (arom.); ^1^H-NMR (CDCl_3_) δ 10.19 (s, 1H, urea), 8.90 (s, 1H, urea), 8.44 (d, *J* = 1.4 Hz, 1H, pyrazine), 8.14–8.04 (m, 2H, pyrazine), 3.44–3.33 (m, 2H, -CH_2_-), 1.70–1.61 (m, 2H, -CH_2_-) 0.99 (t, *J* = 7.4 Hz, 3H, -CH_3_). ^13^C-NMR (CDCl_3_) δ 154.5, 153, 139.5, 137.3, 136.1, 41.3, 22.7, 10.9. Elemental analysis found: C, 53.68%; H, 6.72%; N, 30.89%. Calculated for C_8_H_12_N_4_O (MW 180.21): C, 53.32%; H, 6.71%; N, 31.09%.

*1-Butyl-3-(pyrazin-2-yl)urea* (**11**). Yellow-light brown solid. Yield 29%; m.p. 107.8–108.5 °C; IR (ATR-Ge, cm^−1^): 3276 (-NH-), 2953 (-CH_2_-), 1688 (-C=O), 1600, 1553, 1502 (arom.); ^1^H-NMR (CDCl_3_) δ 10.16 (s, 1H, urea), 8.91 (s, 1H, urea), 8.42 (d, *J* = 1.5 Hz, 1H, pyrazine), 8.14–8.04 (m, 2H, pyrazine), 3.45–3.37 (m, 2H, -CH_2_-), 1.66–1.57 (m, 2H, -CH_2_-), 1.53–1.33 (m, 2H, -CH_2_-), 0.97 (t, *J* = 7.4 Hz, 3H, -CH_3_). ^13^C-NMR (CDCl_3_) δ 156.2, 145, 139.3, 136.4, 136.3, 39.7, 31.9, 20.1, 13.8. Elemental analysis found: C, 55.34%; H, 7.21%; N, 28.31%. Calculated for C_9_H_14_N_4_O (MW 194.24): C, 55.65%; H, 7.27%; N, 28.85%.

*1-Pentyl-3-(pyrazin-2-yl)urea* (**12**). Yellow solid. Yield 51%; m.p. 108–110 °C; IR (ATR-Ge, cm^−1^): 3258 (-NH-), 2955 (-CH_2_-), 1686 (-C=O), 1618, 1599, 1501 (arom.); ^1^H-NMR (CDCl_3_) δ 10.19 (s, 1H, urea), 8.90 (s, 1H, urea), 8.43 (d, *J* = 8.2 Hz, 1H, pyrazine), 8.13–8.04 (m, 2H, pyrazine), 3.44–3.36 (m, 2H, -CH_2_-), 1.44–1.31 (m, 6H, -C_3_H_6_-), 0.92 (t, 3H, -CH_3_). ^13^C-NMR (CDCl_3_) δ 156.2, 150, 139.3, 136.4, 136.3, 40, 29.6, 29.1, 22.3, 14. Elemental analysis found: C, 58.78%; H, 7.95%; N, 26.62%. Calculated for C_10_H_16_N_4_O (MW 208.27): C, 57.67%; H, 7.74%; N, 26.91%.

*1-Octyl-3-(pyrazin-2-yl)urea* (**13**). White solid. Yield 43%; m.p. 115.6–117.2 °C; IR (ATR-Ge, cm^−1^): 3276 (-NH-), 2929 (-CH_2_-), 1699 (-C=O), 1552, 1535, 1503 (arom.); ^1^H-NMR (CDCl_3_) δ 10.21 (s, 1H, urea), 8.91 (s, 1H, urea), 8.43 (d, *J* = 2.8 Hz, 1H, pyrazine), 8.11 (d, *J* = 2.8 Hz, 1H, pyrazine), 8.07 (dd, *J* = 2.8, 1.4 Hz, 1H, pyrazine), 3.40 (td, *J* = 7.1, 5.5 Hz, 2H, -CH_2_-), 1.67–1.57 (m, 4H, -C_2_H_4_-), 1.43–1.37 (m, 2H, -CH_2_-), 1.40–1.26 (m, 4H, -C_2_H_4_-), 1.30–1.21 (m, 2H, -CH_2_-), 0.91–0.84 (m, 3H, -CH_3_).^13^C-NMR (CDCl_3_) δ 156.2, 150, 139.3, 136.3, 136.4, 136.3, 40, 31.8, 29.8, 29.2, 29.1, 27, 22.6, 14. Elemental analysis found: C, 60.39%; H, 8.42%; N, 23.23%. Calculated for C_13_H_22_N_4_O (MW 250.35): C, 60.99%; H, 8.53%; N, 23.71%.

*1-Decyl-3-(pyrazin-2-yl)urea* (**14**). Yellow solid. Yield 24%; m.p. 121.3–123.5 °C; IR (ATR-Ge, cm^−1^): 3270 (-NH-), 2926 (-CH_2_-), 1693 (-C=O), 1600, 1552, 1504 (arom.); ^1^H-NMR (CDCl_3_) δ 10.08 (s, 1H, urea), 8.91 (s, 1H, urea), 8.91 (d, *J* = 2.9 Hz, 1H, pyrazine), 8.91 (d, *J* = 2.9 Hz, 1H, pyrazine), 8.09–8.04 (m, 1H, pyrazine), 3.44–3.36 (m, 2H, -CH_2_-), 1.36–1.21 (m, 16H, -C_8_H_16_-), 0.88 (t, *J* = 6.9 Hz, 3H, -CH_3_). ^13^C-NMR (CDCl_3_) 156.1, 150, 139.3, 136.4, 136.2, 40, 31.9, 29.9, 29.6, 29.5, 29.3, 27, 22.6, 14.1. Elemental analysis found: C, 64.82%; H, 9.37%; N, 19.77%. Calculated for C_15_H_26_N_4_O (MW 278.40): C, 64.71%; H, 9.41%; N, 20.12%.

*1-Benzyl-3-(pyrazin-2-yl)urea* (**15**). Beige solid. Yield 32%; m.p. 176.5–179.3 °C; IR (ATR-Ge, cm^−1^): 3258 (-NH-), 2895 (-CH_2_-), 1694 (-C=O), 1566, 1540, 1508, 1476, 1448 (arom.); ^1^H-NMR (DMSO-*d*_6_) δ 9.51 (s, 1H, urea), 8.91 (s, 1H, urea), 8.26–8.11 (m, 2H, pyrazine), 7.93–7.85 (m, 1H, pyrazine), 7.41–7.27 (m, 5H, arom.), 4.46–4.31 (m, 2H, -CH_2_). ^13^C-NMR (DMSO-*d*_6_) δ 154.8, 150.3, 141.3, 140.1, 137.5, 135.6, 128.9, 127.6, 127.3, 43.2. Elemental analysis found: C, 61.35%; H, 5.30%; N, 24.15%.Calculated for C_12_H_12_N_4_O (MW 228.26): C, 61.67%; H, 5.19%; N, 23.74%.

*1-(4-Methoxyphenyl)-3-(pyrazin-2-yl)urea* (**16**). White solid. Yield 12%; m.p. 225.5–227.4 °C; IR (ATR-Ge, cm^−1^): 3023 (-NH-), 1682 (-C=O), 1614, 1597, 1567, 1551, 1513, 1501 (arom.), 1247 (-C-O); ^1^H-NMR (DMSO-*d*_6_) δ 9.49 (s, 1H, urea), 8.99 (s, 1H, urea), 8.29 (dd, *J* = 2.7, 1.5 Hz, 1H, pyrazine), 8.22 (d, *J* = 2.7 Hz, 2H, pyrazine), 7.44–7.36 (m, 2H, arom.), 6.93–6.86 (m, 2H, arom.), 3.72 (s, 3H, -OCH_3_).^13^C-NMR (DMSO-*d*_6_) δ155.3, 152.0, 149.7, 141.8, 137.8, 135.4, 131.8, 120.9, 114.3, 55.4. Elemental analysis found: C, 59.01%; H, 4.95%; N, 22.14%. Calculated for C_12_H_12_N_4_O_2_ (MW 244.25): C, 60.34%; H, 5.30%; N, 21.97%.

*1-(2-Chlorophenyl)-3-(pyrazin-2-yl)urea* (**17**). White solid. Yield 26%; m.p. 137.5–240.9 °C; IR (ATR-Ge, cm^−1^): 3129 (-NH-), 1699 (-C=O), 1593, 1552, 1509, 1482, 1442 (arom.); ^1^H-NMR (DMSO-*d*_6_) δ 10.35 (s, 1H, urea), 10.28 (s, 1H, urea), 8.85–8.80 (m, 1H, pyrazine), 8.35–8.30 (m, 2H, pyrazine), 7.52–7.46 (m, 2H, arom.), 7.36–7.29 (m, 1H, arom.), 7.11–7.04 (m, 1H, arom.). ^13^C-NMR (DMSO-*d*_6_) δ 151.8, 149.3, 141.1, 137.9, 135.7, 135.6, 129.4, 127.8, 124.2, 122.5, 121.7. Elemental analysis found: C, 53.13%; H, 3.65%; N, 22.53%. Calculated for C_11_H_9_ClN_4_O (MW 248.67): C, 53.22%; H, 3.70%; N, 22.17%.

*1-(4-Chlorophenyl)-3-(pyrazin-2-yl)urea* (**18**). White solid. Yield 29%; m.p. 211–213.7 °C; IR (ATR-Ge, cm^−1^): 3116 (-NH-), 1690 (-C=O), 1603, 1590, 1556, 1504, 1492 (arom.); ^1^H-NMR (DMSO-*d*_6_) δ 9.71 (s, 1H, urea), 9.60 (s, 1H, urea), 9.01 (d, *J* = 1.5 Hz, 1H, pyrazine), 8.30 (dd, *J* = 1.5 Hz, 1H, pyrazine), 8.25 (d, *J* = 1.5 Hz, 1H, pyrazine), 7.60–7.42 (m, 2H, arom.), 7.44–7.27 (m, 2H, arom.). ^13^C-NMR (DMSO-*d*_6_) δ 151.9, 149.4, 141.9, 138.2, 137.9, 135.4, 129.0, 126.6, 120.6. Elemental analysis found: C, 53.13%; H, 3.65%; N, 22.53%. Calculated for C_11_H_9_ClN_4_O (MW 248.67): C, 53.15%; H, 3.61%; N, 22.18%.

*1-(2,3-Dichlorophenyl)-3-(pyrazin-2-yl)urea* (**19**). White solid. Yield 17%; m.p. 246–247.8 °C; IR (ATR-Ge, cm^−1^): 3037 (-NH-), 1734 (-C=O), 1647, 1609, 1593, 1550, 1500 (arom.); ^1^H-NMR (DMSO-*d*_6_) δ 9.89 (s, 1H, urea), 9.67 (s, 1H, urea), 9.06–8.92 (m, 1H, pyrazine), 8.38–8.19 (m, 2H, pyrazine), 7.98–7.84 (m, 1H, arom.), 7.59–7.46 (m, 1H, arom.), 7.42–7.33 (m, 1H, arom.). ^13^C-NMR (DMSO-*d*_6_) δ 151.8, 149.2, 141.8, 139.1, 138.3, 135.4, 131.3, 130.8, 124.3, 120.1, 119.1. Elemental analysis found: C, 46.67%; H, 2.95%; N, 18.19%. Calculated for C_11_H_8_Cl_2_N_4_O (MW 283.11): C, 47.02%; H, 3.42%; N, 17.64%.

*1-(2-Chlorobenzyl)-3-(pyrazin-2-yl)urea* (**20**). White solid. Yield 33%; m.p. 204.6–207.6 °C; IR (ATR-Ge, cm^−1^): 3240 (-NH-), 1681(-C=O), 1567, 1580, 1542, 1505 (arom.); ^1^H-NMR (DMSO-*d*_6_) δ 9.59 (s, 1H, urea), 8.88 (s, 1H, urea), 8.27–8.12 (m, 2H, pyrazine), 7.99 (t, *J* = 5.9 Hz, 1H, pyrazine), 7.50–7.25 (m, 4H, arom.). ^13^C-NMR (DMSO-*d*_6_) 154.7, 150.2, 141.8, 137.6, 137.1, 135.5, 132.6, 129.7, 129.4, 129.2, 127.8. Elemental analysis found: C, 54.87%; H, 4.22%; N, 21.33%. Calculated for C_12_H_11_ClN_4_O (MW 262.70): C, 54.98%; H, 4.15%; N, 21.35%.

### 3.4. Biological Assays

#### 3.4.1. In Vitro Activity Evaluation against *Mycobacterium tuberculosis*, *Mycobacterium kansasii*, and *Mycobacterium avium*

Microdilution panel method. Tested strains *M. tuberculosis* H37Rv CNCTC My 331/88 (ATCC 27294), *M. kansasii* Hauduroy CNCTC My 235/80 (ATCC 12478), *M. avium* ssp. *Avium* Chester CNCTC My 80/72 (ATCC 15769) were obtained from Czech National Collection of Type Cultures (CNCTC), National Institute of Public Health, Prague, Czech Republic. Middlebrook 7H9 broth (Sigma-Aldrich) enriched with 0.4% (*v*/*v*) of glycerol (Sigma-Aldrich) and 10% (*v*/*v*) of OADC supplement (oleic acid, albumin, dextrose, catalase; Himedia, Mumbai, India) of declared pH = 6.6. Tested compounds were dissolved and diluted in DMSO, mixed with broth (25 µL) of DMSO solution in 4.475 mL of broth and placed (100 µL) into microplate wells. Mycobacterial inocula were suspended in isotonic saline solution and the density was adjusted to 0.5–1.0 McFarland scale. These suspensions were diluted by 10^−1^ and used to inoculate the testing wells, adding 100 µL of mycobacterial suspension per well. Final concentrations of the tested compounds in wells were 100, 50, 25, 12.5, 6.25, 3.13, and 1.56 µg/mL. INH and PZA were used as positive controls (inhibition of growth). Negative control (mycobacterial growth control) consisted of broth plus DMSO. Plates were statically incubated in a dark, humid atmosphere at 37 °C. After five days of incubation, 30 µL of Alamar Blue working solution (1:1 mixture of 0.1% resazurin sodium salt (aq. sol.) and 10% Tween 80) was added per well. Results were then determined after 24 h of incubation and interpreted according to Franzblau et al. [21]. The minimum inhibition concentration (MIC, μg/mL) was determined as the lowest concentration that prevented the blue to pink colour change as indicated by visual inspection. The experiments were conducted in duplicates. For the results to be valid, the difference in MIC for one compound determined from two parallel measurements must not be greater than one step on the dilution scale.

#### 3.4.2. In Vitro Activity Evaluation against *Mycobacterium smegmatis* and *Mycobacterium aurum*

Antimycobacterial assay was performed on fast growing *M. smegmatis* DSM 43465 (ATCC 607) and *M. aurum* DSM 43999 (ATCC 23366) from German Collection of Microorganisms and Cell Cultures (Braunschweig, Germany). The technique used for activity determination was microdilution broth panel method using 96-well microtitration plates. Culturing medium was Middlebrook 7H9 broth (Sigma-Aldrich) enriched with 0.4% of glycerol (Sigma-Aldrich) and 10% of Middlebrook OADC growth supplement (Himedia). Mycobacterial strains were cultured on Middlebrook 7H9 agar and suspensions were prepared in Middlebrook 7H9 broth. Final density was adjusted to value ranging from 0.5 to 1.0 according to McFarland scale and diluted in ratio 1:20 with broth. Tested compounds were dissolved in DMSO (Sigma-Aldrich) then MB broth was added to obtain concentration of 2000 µg/mL. Standards used for activity determination were INH, rifampicin (RIF) and ciprofloxacin (CPX) (Sigma-Aldrich). Final concentrations were reached by binary dilution and addition of mycobacterial suspension, and were set as 500, 250, 125, 62.5, 31.25, 15.625, 7.81, 3.91 µg/mL, except to standards rifampicin, where the final concentrations were 12.5, 6.25, 3.125, 1.56, 0.78, 0.39, 0.195, 0.098 µg/mL, and ciprofloxacin, where the final concentrations were 1, 0.5, 0.25, 0.125, 0.0625, 0.0313, 0.0156, 0.0078 µg/mL. The final concentration of DMSO did not exceeded 2.5% (*v*/*v*) and did not affect the growth of *M. smegmatis* or *M. aurum*. Positive (broth, DMSO, bacteria) and negative (broth, DMSO) controls were included. Plates were sealed with polyester adhesive film and incubated in dark at 37 °C without agitation. The addition of 0.01% solution of resazurin sodium salt followed after 48 h of incubation for *M. smegmatis*, and after 72 h of incubation for *M. aurum*. Stain was prepared by dissolving resazurin sodium salt (Sigma-Aldrich) in deionised water to get 0.02% solution. Then 10% aqueous solution of Tween 80 (Sigma-Aldrich) was prepared. Equal volumes of both liquids were mixed and filtered a through syringe membrane filter. Microtitration panels were then incubated for further 2.5 h for determination of activity against *M. smegmatis*, and 4 h for *M. aurum*. Antimycobacterial activity was expressed as minimal inhibition concentration (MIC) and the value was read on the basis of stain colour change (blue colour—active compound; pink colour—inactive compound). MIC values for standards were in ranges 7.81–15.625 µg/mL for INH, 12.5–25 µg/mL for RIF, and 0.0625–0.125 µg/mL for CPX against *M. smegmatis*, 1.95–3.91 µg/mL for INH, 0.78–1.56 µg/mL for RIF, and 0.00781–0.01563 µg/mL for CPX against *M. aurum*, respectively. All experiments were conducted in duplicate. For the results to be valid, the difference in MIC for one compound determined from two parallel measurements must not be greater than one step on the dilution scale.

#### 3.4.3. In Vitro Antibacterial Activity Evaluation

Microdilution broth method was used [35]. Antibacterial evaluation was performed against eight bacterial strains from the Czech Collection of Microorganisms (CCM, Brno, Czech Republic) (*Staphylococcus aureus* CCM 4223 (ATCC 29213), *Staphylococcus aureus* methicilin resistant CCM 4750 (ATCC 43300), *Enterococcus faecalis* CCM 4224 (ATCC 29212), *Escherichia coli* CCM 3954 (ATCC 25922), *Pseudomonas aeruginosa* CCM 3955 (ATCC 27853)) or clinical isolates from the Department of Clinical Microbiology, University Hospital and Faculty of Medicine in Hradec Králové, Charles University in Prague, Czech Republic (*Staphylococcus epidermidis* 112-2016, *Klebsiella pneumoniae* 64-2016, *Serratia marcescens* 62-2016). All strains were subcultured on Mueller-Hinton agar (MHA) (Difco/Becton Dickinson, Detroit, MI, USA) at 35 °C and maintained on the same medium at 4 °C. The compounds were dissolved in DMSO, and the antibacterial activity was determined in cation adjusted Mueller-Hinton liquid broth (Difco/Becton Dickinson) buffered to pH 7.0. Controls consisted of medium and DMSO solely. The final concentration of DMSO in the test medium did not exceed 1% (*v*/*v*) of the total solution composition. The minimum inhibitory concentration (MIC) was determined after 24 and 48 h of static incubation at 35 °C by visual inspection or using Alamar Blue dye. The standards were gentamicin and ciprofloxacin. All experiments were conducted in duplicate. For the results to be valid, the difference in MIC for one compound determined from two parallel measurements must not be greater than one step on the dilution scale.

#### 3.4.4. In Vitro Antifungal Activity Evaluation

Antifungal evaluation was performed using a microdilution broth method [29] against eight fungal strains from the Czech Collection of Microorganisms (CCM) (*Candida albicans* CCM 8320 (ATCC 24433), *C. krusei* CCM 8271 (ATCC 6258), *C. parapsilosis* CCM 8260 (ATCC 22019), *C. tropicalis* CCM 8264 (ATCC 750), *Aspergillus flavus* CCM 8363, *Absidia/Lichtheimia corymbifera* CCM 8077 and *Trichophyton interdigitale* CCM 8377 (ATCC 9533) or the American Type Collection Cultures (ATCC, Mannasas, VA, USA) (*Aspergillus fumigatus* ATCC 204305). Compounds were dissolved in DMSO and diluted in a twofold manner with RPMI 1640 medium, with glutamine and 2% glucose, buffered to pH 7.0 (3-morpholinopropane-1-sulfonic acid). The final concentration of DMSO in the tested medium did not exceed 2.5% (*v*/*v*) of the total solution composition. Static incubation was performed in the dark and in humid atmosphere, at 35 °C, for 24 and 48 h (72 and 120 h for *Trichophyton interdigitale* respectively). Drug-free controls were included. MIC was inspected visually or making use of Alamar Blue staining. The standards were amphotericin B and fluconazole. All experiments were conducted in duplicate. For the results to be valid, the difference in MIC for one compound determined from two parallel measurements must not be greater than one step on the dilution scale.

#### 3.4.5. Cytotoxicity Determination

Human hepatocellular liver carcinoma cell line HepG2 (passage 9) purchased from Health Protection Agency Culture Collections (ECACC, Salisbury, UK) was cultured in MEM (Minimum Essentials Eagle Medium) (Sigma-Aldrich, St. Louis, MO, USA) supplemented with 10% fetal bovine serum (PAA), 1% l-Glutamine solution (Sigma-Aldrich) and non-essential amino acid solution (Sigma-Aldrich) in a humidified atmosphere containing 5% CO_2_ at 37 °C. For subculturing, the cells were harvested after trypsin/EDTA (Sigma-Aldrich) treatment at 37 °C. To evaluate cytotoxicity, the cells treated with the tested substances were used as experimental groups whereas untreated HepG2 cells served as controls. The cells were seeded in density of 10,000 cells per well in a 96-well plate. On the following day, the cells were treated with each of the tested substances dissolved in DMSO. The tested substances were prepared at different incubation concentrations (Table 2) in triplicates according to their solubility. Simultaneously, the controls representing 100% cell viability, 0% cell viability (the cells treated with 10% DMSO), no cell control and vehiculum controls, were also prepared in triplicates. After 24 h incubation in a humidified atmosphere containing 5% CO_2_ at 37%, the reagent from the kit CellTiter 96 Aqueous One Solution Cell Proliferation Assay (CellTiter 96; PROMEGA, Fitchburg, MA, USA) was added. After 2 h incubation at 37%, absorbance of samples was recorded at 490 nm (TECAN, Infinita M200, Sydney, Austria). A standard toxicological parameter IC_50_ was calculated by nonlinear regression from a semilogarithmic plot of incubation concentration versus percentage of absorbance relative to untreated controls using GraphPad Prism 7 software (GraphPad Software, La Jolla, CA, USA).

#### 3.4.6. Plant Growth Regulation Activity Evaluation

Callus cultures were derived from germinating seeds of *Fagypyrum esculentum* var. Bamby. Seeds were obtained from Crop Research Institute (Piešťany, Slovak Republic). *F. esculentum* callus cultures in the 23–25th passages were used. Calluses were cultivated on Murashige-Skoog medium [36] supplemented with 2,4-dichlorophenoxyacetic acid (2,4-D) at a concentration of 1 mg/L as growth regulator. Callus cultures were cultivated on paper bridges in Erlenmeyer flasks for 4 weeks in growth chambers at 26 ± 1 °C for 16 h photoperiod. White light intensity of 3.500 lux was used.

The abiotic elicitors, **8** at the concentration of 2.993 × 10^−3^ mol/L and **18** at a concentration of 4.056 × 10^−3^ mol/L, were tested. Proposed elicitors (1 mL) were added to the callus cultures on the 21st day of cultivation. After 6, 12, 24, 48, 72, and 168 h of elicitor treatment the calluses were sampled and dried, and then the content of rutin was determined. Simultaneously, controls without elicitor application were run after 24 and 168 h. Rutin content was estimated according to Kreft et al. [37]. All experiments were conducted in triplets.

## 4. Conclusions

In this research project we prepared eighteen different ureidopyrazine derivatives. We focused on testing the anti-infective activity of the prepared compounds against *Mycobacterium tuberculosis* and five other nontubercular strains, along with antibacterial and antifungal activity evaluation. We found five compounds active against *Mtb* out of the twenty tested, and we have started an initial structure-activity relationships (SAR) study. According to the results of antimycobacterial assays, the two most active compounds, compound **4** (MIC*_Mtb_* = 1.56 μg/mL, 5.19 μM) and compound **6** (MIC*_Mtb_* = 6.25 μg/mL, 18.91 μM), were aryl substituted ureidopyrazine propyl esters. Those two compounds were proven to be nontoxic on HepG2 cancer cells. All tested compounds had neither antibacterial nor antifungal activity. Two of the prepared compounds, compounds **8** and **18**, structurally resemble a known plant growth regulator and other pyrazinecarboxamides that were proved to be abiotic elicitors. Those two compounds were assessed for similar activity and were found to be promising elicitors by stimulating rutin production in plant cultures. Compounds **4** and **6** can be possible starting points for future research that can improve both biological activity and physicochemical properties. Their anti-TB activity will be further evaluated against resistant strains of *Mtb* and their mechanism of action shall be investigated.

## Figures and Tables

**Table 1:** Structure of prepared compounds, antimycobacterial activity expressed by minimum inhibitory concentrations (MIC), and cytotoxicity expressed by IC_50_ values.

A		B		C & D		
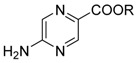		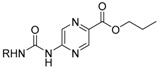		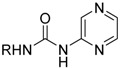		
	No.	R	log *P*	Antimycobacterial Activity MIC (μg/mL)		
				*Mtb*	*M. kansasii*	*M. avium*
A	**1**	H	−0.75	>100	>100	>100
	**2**	propyl	0.34	>100	>100	>100
B	**3**	benzyl	1.69	>100	>100	>100
	**4**	phenyl	1.62	1.56	>100	>100
	**5** ^a^	phenyl	0.54	>100	>100	>100
	**6**	4-methoxyphenyl	1.50	6.25	25	>100
	**7**	2-chlorophenyl	2.18	>100	>100	>100
	**8**	4-chlorophenyl	2.18	25	>100	>100
	**9**	3,4-dichlorophenyl	2.74	>100	>100	>100
C	**10**	propyl	−0.28	>100	>100	>100
	**11**	butyl	0.13	>100	>100	>100
	**12**	pentyl	0.55	100	>100	>100
	**13**	octyl	1.8	25	25	>100
	**14**	decyl	2.64	>100	>100	>100
D	**15**	benzyl	0.62	>100	>100	>100
	**16**	4-methoxyphenyl	0.43	>100	>100	>100
	**17**	2-chlorophenyl	1.11	>100	>100	>100
	**18**	4-chlorophenyl	1.11	12.5	>100	>100
	**19**	3,4-dichlorophenyl	1.67	>100	>100	>100
	**20**	2-chlorobenzyl	0.14	>100	>100	>100
	PZA		−1.31	>100 ^b^	>100	>100
	INH		−0.64	0.2–0.4	6.25–12.5	6.25–12.5

^a^ Free acid form of compound **4** prepared by base catalyzed hydrolysis; ^b^ MIC value from testing at pH = 5.6 (acidic) is 6.25–12.5 μg/mL [22]. The value stated in the table is from testing at pH = 6.6 (neutral).

**Table 2:** Cytotoxicity of the tested substances in HepG2 cells.

Compound	IC_50_ (μM)
**4**	>25 * (>16 × MIC*_Mtb_*)
**6**	>50 * (>8 × MIC*_Mtb_*)
PZA	>10^4^ [31]
INH	79 × 10^3^ [31]

* Measurement at higher concentrations was not possible due to the precipitation of the tested compounds in cell culture medium.

## Supplementary Materials

Supplementary materials are available online at www.mdpi.com/1420-3049/22/10/1797/s1. Table S1: Structure of prepared compounds with their activity against fast growing *M. smegmatis* and *M. aurum*, Table S2: Antibacterial assay results of prepared compounds, Table S3: Antifungal assay results of prepared compounds, Table S4: Rutin content (µg g^−1^ DW) in *Fagopyrum esculentum* var. Bamby callus culture after treatment with compounds **8** and **18**, ^1^H- and ^13^C-NMR spectra of the most active compound **4**.
