# Supplementary material for: Ureidopyrazine Derivatives: Synthesis and Biological Evaluation as Anti-Infectives and Abiotic Elicitors
Source: Molecules. 2017 Oct 23;22(10):1797. doi: 10.3390/molecules22101797 (PMC6151446; doi:10.3390/molecules22101797)

Ureidopyrazine Derivatives: Synthesis and Biological Evaluation as Anti-infectives and Abiotic Elicitors

Ghada Bouz*, Martin Juhás, Pavlína Niklová, Ondřej Janďourek, Pavla Paterová, Jiří Janoušek, Lenka Tůmová, Zuzana Kovalíková, Petr Kastner, Martin Doležal, Jan Zitko*.

Faculty of Pharmacy in Hradec Kralove, Charles University, Heyrovskeho 1203, Hradec Kralove 50005, Czech Republic; e-mails: juhasm@faf.cuni.cz (M.J.); niklovp@faf.cuni.cz (P.N.); JANDO6AA@faf.cuni.cz (O.J.); pavla.paterova@fnhk.cz (P.P.); janousj2@faf.cuni.cz (J.J.); lenka.tumova@faf.cuni.cz (L.T.); kovalikz@faf.cuni.cz (Z.K.); kastner@faf.cuni.cz (P.K.); dolezalm@faf.cuni.cz (M.D.).

* Correspondence: e-mails: bouzg@faf.cuni.cz (G.B.); jan.zitko@faf.cuni.cz (J.Z.); Tel.: +420-495-067-275 (G.B.); +420-495-067-272 (J.Z.).

Supplementary Materials

Table S1: Prepared compounds with their activity against fast growing *M. smegmatis* and *M. aurum*.

No.	R	Antimycobacterial Activity MIC (μg/mL)	
		M. smeg	*M. aurum*
1	hydrogen	>500	>500
2	propyl	>500	>500
3	benzyl	≥500	≥500
4	phenyl	≥500	≥500
5	phenyl	≥500	≥500
6	4-methoxyphenyl	≥500	≥500
7	2-chlorophenyl	≥500	≥500
8	4-chlorophenyl	≥250	≥250
9	3,4-dichlorophenyl	≥125	≥125
10	propyl	≥500	≥500
11	butyl	≥500	250
12	pentyl	≥500	125
13	octyl	≥500	≥500
14	decyl	≥250	≥250
15	benzyl	≥500	≥500
16	4-methoxyphenyl	≥500	≥500
17	2-chlorophenyl	≥125	≥125
18	4-chlorophenyl	≥500	≥500
19	3,4-dichlorophenyl	≥250	≥250
20	2-chlorobenzyl	≥125	≥125
	INH	7.81-15.63	1.95-3.91
	RFM	12.5-25	0.78-1.56
	CPX	0.06-0.13	0.008-0.016

Table S2: Antibacterial assay results of prepared compounds.

Pathogen	Time(h)	Antibacterial Activity [MIC (μmol.l^{-1})]														
		**1**	**2**	**4**	**5**	**8**	**10**	**11**	**12**	**13**	**14**	**15**	**16**	**17**	**19**	**20**
**SA**	**24hr**	>500	>500	>125	>500	>125	>500	>500	>500	>500	>250	>500	>125	>500	>500	>500
	**48hr**	>500	>500	>125	>500	>125	>500	>500	>500	>500	>250	>500	>125	>500	>500	>500
**MRSA**	**24hr**	>500	>500	>125	>500	>125	>500	>500	>500	>500	>250	>500	>125	>500	>500	>500
	**48hr**	>500	>500	>125	>500	>125	>500	>500	>500	>500	>250	>500	>125	>500	>500	>500
**SE**	**24hr**	>500	>500	>125	>500	>125	>500	>500	>500	>500	>250	>500	>125	>500	>500	>500
	**48hr**	>500	>500	>125	>500	>125	>500	>500	>500	>500	>250	>500	>125	>500	>500	>500
**EF**	**24hr**	>500	>500	>125	>500	>125	>500	>500	>500	>500	>250	>500	>125	>500	>500	>500
	**48hr**	>500	>500	>125	>500	>125	>500	>500	>500	>500	>250	>500	>125	>500	>500	>500
**EC**	**24hr**	>500	>500	>125	>500	>125	>500	>500	>500	>500	>250	>500	>125	>500	>500	>500
	**48hr**	>500	>500	>125	>500	>125	>500	>500	>500	>500	>250	>500	>125	>500	>500	>500
**KP**	**24hr**	>500	>500	>125	>500	>125	>500	>500	>500	>500	>250	>500	>125	>500	>500	>500
	**48hr**	>500	>500	>125	>500	>125	>500	>500	>500	>500	>250	>500	>125	>500	>500	>500
**SEMA**	**24hr**	>500	>500	>125	>500	>125	>500	>500	>500	>500	>250	>500	>125	>500	>500	>500
	**48hr**	>500	>500	>125	>500	>125	>500	>500	>500	>500	>250	>500	>125	>500	>500	>500
**PA**	**72hr**	>500	>500	>125	>500	>125	>500	>500	>500	>500	>250	>500	>125	>500	>500	>500
	**120hr**	>500	>500	>125	>500	>125	>500	>500	>500	>500	>250	>500	>125	>500	>500	>500

1. **SA**- *Staphylococcus aureus*
2. **MRSA**- *Staphylococcus aureus* methicilin resistant
3. **SE**- *Staphylococcus epidermidis*
4. **EF**- *Enterococcus faecalis*
5. **EC**- *Escherichia coli*
6. **KP**- *Klebsiella pneumoniae*
7. **SEMA**- *Serratia marcescens*
8. **PA**- *Pseudomonas aeruginosa*

Table S3: Antifungal assay results of prepared compounds.

Pathogen	Time(h)	Antifungal Activity [MIC ($\mu mol.l^{-1}$)]														
		1	2	4	5	8	10	11	12	13	14	15	16	17	19	20
CA	24hr	>500	>500	>125	>500	>125	>500	>500	>500	>500	>250	>500	>125	>500	>500	>500
	48hr	>500	>500	>125	>500	>125	>500	>500	>500	>500	>250	>500	>125	>500	>500	>500
CK	24hr	>500	>500	>125	>500	>125	>500	>500	>500	>500	>250	>500	>125	>500	>500	>500
	48hr	>500	>500	>125	>500	>125	>500	>500	>500	>500	>250	>500	>125	>500	>500	>500
CP	24hr	>500	>500	>125	>500	>125	>500	>500	>500	>500	>250	>500	>125	>500	>500	>500
	48hr	>500	>500	>125	>500	>125	>500	>500	>500	>500	>250	>500	>125	>500	>500	>500
CT	24hr	>500	>500	>125	>500	>125	>500	>500	>500	>500	>250	>500	>125	>500	>500	>500
	48hr	>500	>500	>125	>500	>125	>500	>500	>500	>500	>250	>500	>125	>500	>500	>500
AF	24hr	>500	>500	>125	>500	>125	>500	>500	>500	>500	>250	>500	>125	>500	>500	>500
	48hr	>500	>500	>125	>500	>125	>500	>500	>500	>500	>250	>500	>125	>500	>500	>500
AFla	24hr	>500	>500	>125	>500	>125	>500	>500	>500	>500	>250	>500	>125	>500	>500	>500
	48hr	>500	>500	>125	>500	>125	>500	>500	>500	>500	>250	>500	>125	>500	>500	>500
AC	24hr	>500	>500	>125	>500	>125	>500	>500	>500	>500	>250	>500	>125	>500	>500	>500
	48hr	>500	>500	>125	>500	>125	>500	>500	>500	>500	>250	>500	>125	>500	>500	>500
TI	72hr	>500	>500	>125	>500	>125	>500	>500	>500	>500	>250	>500	>125	>500	>500	>500
	120hr	>500	>500	>125	>500	>125	>500	>500	>500	>500	>250	>500	>125	>500	>500	>500

1. **CA1**- *Candida albicans*

2. **CK**- *Candida krusei*

3. **CP**- *Candida parapsilosis*

4. **CT**- *Candida tropicalis*

5. **AF**- *Aspergillus fumigatus*

6. **AFla**- *Aspergillus flavus*

7. **AC**- *Absidia/Lichtheimia corymbifera*

8. **TI**- *Trichophyton interdigitale*

Table S4: Rutin content (μg.g^{-1} DW) in *Fagopyrum esculentum* var. Bamby callus culture after treatment with compounds 8 and 18.

Time (h)	Rutin content (μg.g^{-1} DW) for compound 8 at conc. 2.993.10^{-3} mol.l^{-1}	Rutin content (μg.g^{-1} DW) For compound 18 at conc. 4.056. 10^{-3} mol.l^{-1}
6	0.80	1.01
12	0.83	0.53
24	0.58	0.57
24K*	0.47	0.47
48	0.27	0.21
72	0.08	0.36
168	0.00	0.00
168K*	0.00	0.21

*K: control with no elicitor

^{1}HNMR spectra for compound 4

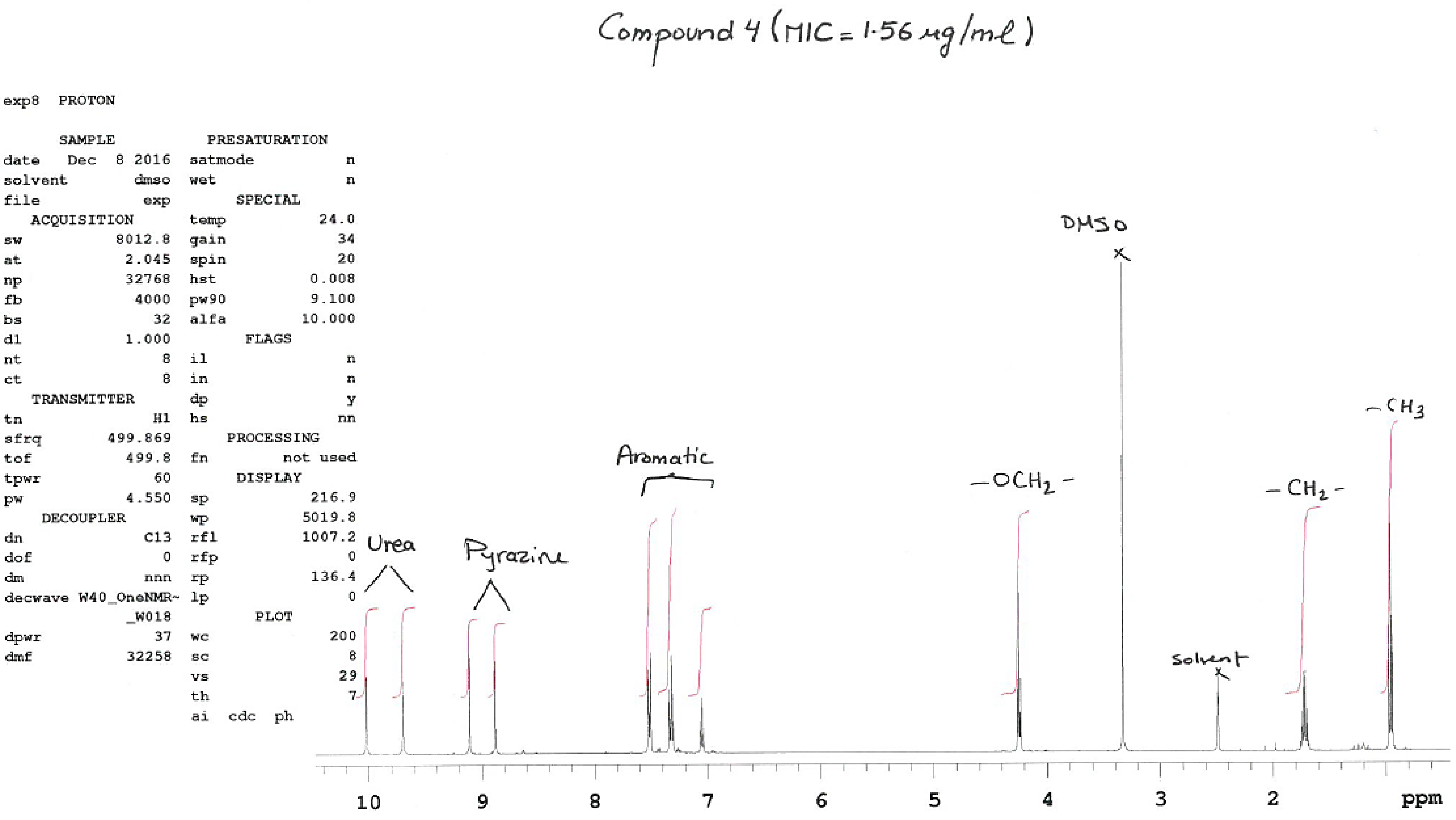

^{13}CNMR for compound 4

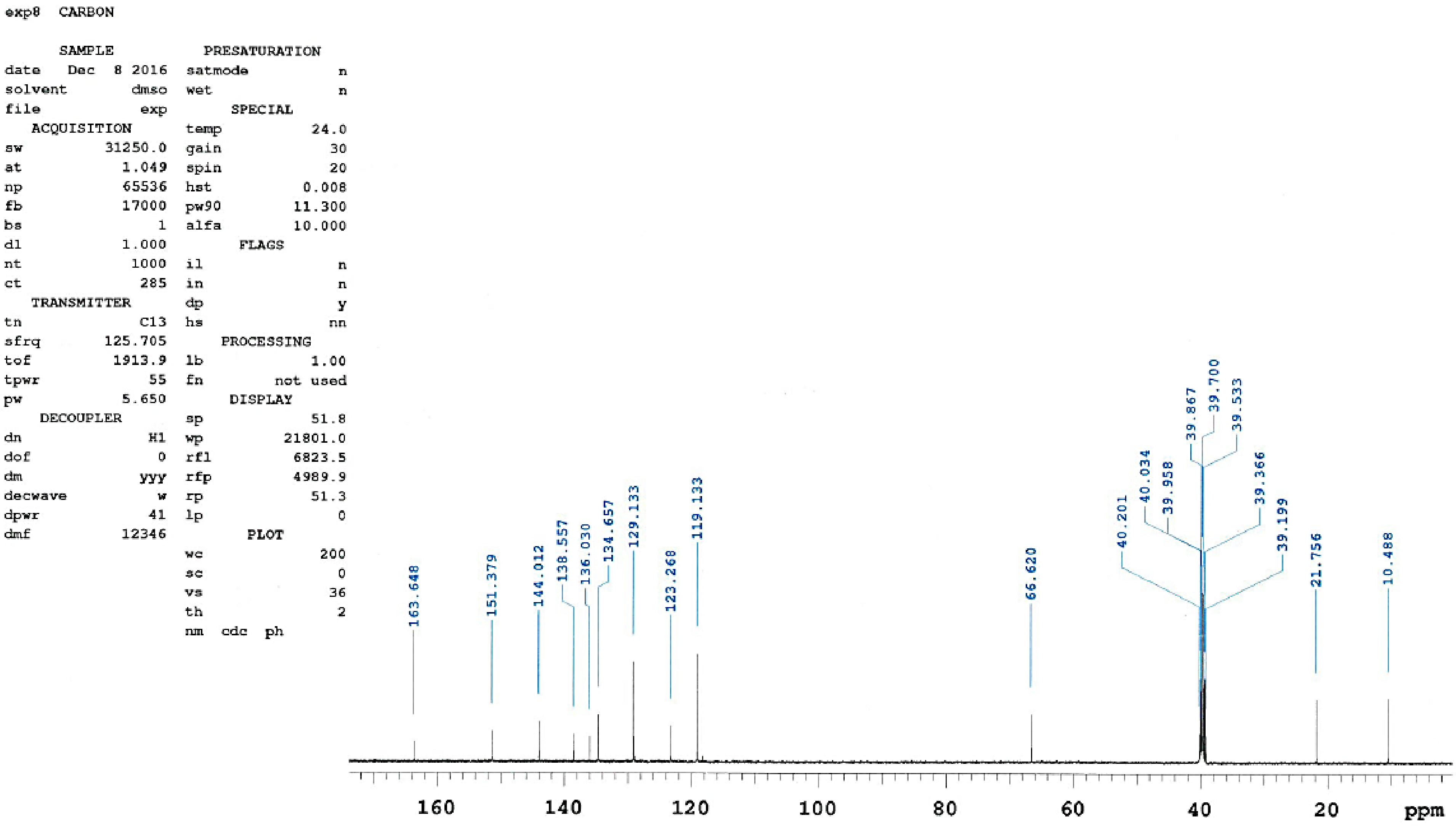

Supplement: Supplementary file 1 [file molecules-22-01797-s001.pdf]
